# Surgical and Pathological Cases

**Published:** 1832-01

**Authors:** James Laidlaw

**Affiliations:** Member of the Royal Colleges of Surgeons of London and Edinburgh, and formerly House Surgeon to the Middlesex Hospital.


### SURGICAL AND PATHOLOGICAL CASES.

*Surgical and Pathological Cases.* By JAMES LAIDLAW, Esq., Member of the Royal Colleges of Surgeons of London and Edinburgh, and formerly House Surgeon to the Middlesex Hospital.

(Continued from page 458 of the last volume.)

### *Of Retention of Urine, and Stricture of the Urethra.*

IT frequently happens, and more particularly in hospital practice, that a number of cases of retention of urine require the attention of the surgeon nearly at the same time, (that is to say, within the course of a few hours;) while, at other times, an entire month will elapse without the occurrence of a single case. During the period I resided at the Middlesex Hospital as house surgeon, this fact so repeatedly happened as to direct my attention to a careful consideration of it; and I came to the conclusion that it could not be the result of mere accident, but must, in a great measure, depend upon the state of the atmosphere, especially as by far the greater number of the cases thus occurring together were cases in which the retention was entirely caused by spasm; and although in several of them stricture was found, yet the patients stated that they were seldom inconvenienced by it, as they could always pass their water in a stream, except when they had been drinking or had caught cold, and then (to use their own words) they were seized with the spasms. Now, notwithstanding it frequently happens that a man, having an irritable bladder and urethra, and perhaps a stricture, by incautiously exposing himself to severe cold or wet, brings on retention of urine, yet it cannot be easily conceived that there should occur so singular a coincidence as that six or eight persons, under the same circumstances, should, by their carelessness and inattention, expose themselves at the same time to the risk of an attack of so alarming a complaint as retention of urine; or that they should all apply at the same place for relief, and that within a very short time of one another. In fact, the patients themselves seemed to be perfectly aware of the influence of the weather in producing their distress, as they have frequently replied, when asked if they have many attacks of the complaint, "No, only sometimes during this sort of weather, or upon catching cold." Another circumstance confirmatory of the truth of the above observations is the great facility with which all such cases were relieved, (although in many of them the inability of passing water had existed upwards of twelve hours,) by merely placing the patient in a warm bed, fomenting the abdomen and perineum, and by the administration of an opiate: the patients soon expressed their relief, and generally in less than half an hour a catheter could be got into the bladder, without difficulty. As cases of this nature occurred not unfrequently, I kept memoranda of them when any unusual number required my attention on the same day: perhaps the following extract from my case-book may not be uninteresting, as illustrative of the above remarks.

March 30th, 1829. During yesterday and today, the weather has changed from that of a fine, mild, and spring-like atmosphere, to a dull hazy state, with a north-west wind, and occasionally a drizzling rain: it seems to produce in many persons an unusual irritability of the bladder and urethra; as, within the last twenty-four hours, seven persons have applied at the hospital for relief, in consequence of inability to pass their water, and one patient, in the house for another complaint, has had an attack of retention.

The first applicant, John Morgan, had been under treatment for stricture some time back; his urethra would admit a large catheter to pass into the bladder easily, but, as a matter of precaution, he was recommended to have a bougie passed occasionally: he has had, till this morning no inability of making water for a long time; he had not been exposed to cold or wet, and discovered the complaint soon after he rose in the morning, and came here immediately, when, with very little difficulty, I relieved him by the introduction of a catheter, and gave him a dose of castor oil, with a few drops of laudanum, and dismissed him.

The next patient applied in the afternoon: his inability to make water had come on eight hours previous to his application at the hospital, during which time he had not been able to void a drop. He stated that it was two years since he had had a similar attack, when he was relieved by a surgeon in the country, who, after repeated trials, succeeded in introducing a catheter. He always made water in a full stream when free from these attacks. A good-sized gum elastic catheter was introduced without difficulty, and, after warning him not to expose himself to cold, he was sent home.

Two hours after his dismission, a young fellow, apparently in great distress, applied to me, stating that he had not made water for many hours, and was exceedingly anxious to have a catheter passed, as he had been relieved by it on a former occasion, when he had retention of urine from gonorrhœa; since which time (eight months ago,) he had always passed his water in a full stream, and without the slightest difficulty. I made use of the same catheter as in the former cases, with the same success, and made him an out-patient.

In the course of the evening three other cases occurred, so precisely similar to the above, in every important feature, that an account of them would be a mere repetition; they were all shortly relieved by the catheter, and it was not found necessary to admit them into the hospital, but they were furnished with medicine as out-patients, and directed to return if they should find it necessary.

About eight o'clock another case occurred: John Styles, aged thirty; he had had total retention of urine since three o'clock in the morning, that is, for a period of seventeen hours.

He stated that he had been occasionally subject to the complaint for upwards of seven years, and thought it arose from gonorrhœa; he had been repeatedly relieved during that period by the catheter, and his medical attendants told him he had *several* strictures: he thought his present attack arose from his having been exposed to cold and wet. When I first saw him, he presented one of the most miserable and pitiable objects imaginable: his countenance was anxious and expressive of great misery; he had been running from one chemist's shop to another, seeking relief, and during the day many attempts had been made to pass a catheter, which had only increased the irritation. In addition to all this misery, he was completely wetted through by running about in the rain, and he said that latterly he had been half starved, in consequence of want of employment. Upon examining him, I found the bladder risen above the pubis, and, upon the most gentle attempt to pass a bougie, the urethra bled copiously, and he described the pain as very severe, though he was willing to submit to any thing for relief.

He was instantly placed in a warm bed; leeches were applied to the perineum, and hot fomentations to the abdomen, and some castor oil, with forty drops of laudanum, was given him. He soon expressed his relief, saying the fomentations were very comfortable; and as the case was very urgent, and the warm bath could not be got ready for some time, I determined to attempt getting an instrument into the bladder. After persevering for more than half an hour with the finest elastic gum bougie, I felt it firmly grasped in the stricture, and allowed it to remain so for some time, continuing the application of the fomentations; I then gently urged it forward, and succeeded in passing it into the bladder: after waiting a short time, it was carefully withdrawn, in hopes the urine would follow, but not a drop flowed. Being thus provokingly disappointed, I introduced the fine bougie I had withdrawn into a small catheter, so as to serve as a sort of flexible stilet to it, and thus it gained much greater strength without losing its elasticity. This I succeeded in getting into the stricture, and, finding it quite firm, I pushed it on with considerable force, and got it into the bladder. I immediately withdrew the bougie, and the urine flowed through the catheter; but, as the instrument was very small, it was a considerable time before a pint was passed; and, when he had passed rather more than that quantity, he was so much relieved, and at the same time so overcome by his previous fatigue and distress, that, without waiting to empty the bladder, he threw himself down in the bed, and was fast asleep in a minute or two: the urine continued to dribble away through the catheter for some time.

In the morning the instrument was found completely choked up with mucus, and no urine could flow through it: he could, however, pass the urine by the side of the instrument; and, the spasm having subsided, the catheter was withdrawn, and he then made water quite freely, and in a full stream.

The above were the greatest number of cases I remember to have seen in so short a space of time; but it was by no means uncommon for several to occur in the above marked manner in the course of twenty-four hours. I never hesitated to attempt the instant introduction of a catheter, and was in general successful, although I believe it is generally considered more judicious to endeavour to allay the irritable state of the urethra, by anodynes, fomentations, and the warm bath, prior to any mechanical attempts, which are supposed only to increase the irritation. But, however good the latter theory may be, I believe it is generally found to be impracticable, as the patients are well aware of the relief to be expected from the introduction of an instrument, and if an attempt to pass one is not made immediately, they will in general seek other assistance: neither do I consider that an attempt to pass the catheter does, in the majority of cases, produce increased irritation; that is to say, if the instrument be handled with skill and dexterity, and be slid, as it were, without the exertion of force along the urethra. In by far the greater number of cases, the patient will not complain of pain, unless force be employed, or the urethra has been injured by previous attempts. Another circumstance justifying the immediate use of the instrument, is the ignorance of the urgency of the case under which the surgeon must frequently labour, inasmuch as one man, whose bladder will hold several pints, may have the complaint for several days without being in much danger; while another shall have a bladder so small and diseased, that ulceration will take place from the retention, for a few hours only, of less than half a pint of urine. This last-mentioned circumstance must be familiar to every one who has had opportunities of seeing preparations of morbid anatomy: I think, in one of the collections which belonged to Sir Charles Bell, I remember many such preparations, in which the bladders, at their utmost state of distention, could not have held more than a few ounces: indeed, their walls were more like those of a uterus than of a bladder. In such cases, of course, it would be impossible to judge of the urgency of the case (as directed by many writers,) by feeling for the bladder above the pubis: it would burst before it would rise high enough to be felt there. I was lately called to a case of the above nature; the retention of urine was of only a few hours' continuance, but I found my patient in very great distress, and slightly delirious from the irritation: he had been long subject to the complaint, and had been in the habit of using fomentations and taking opiates with ether, &c. whenever his attacks came on. It was evident his life was to be saved only by the instant introduction of a catheter, or by puncturing the bladder: fortunately I succeeded in passing the instrument, and then found that all this alarming distress had been caused by the retention of less than half a pint of urine.

*Retention of Urine, succeeded by Ulceration of the Urethra and Bladder, with Abscess of the Prostate Gland.*

George Flemming, twenty-eight years of age, a subaltern on half-pay in the East India Company's service, was brought to the Middlesex Hospital, on the 12th May, 1829, labouring under extravasation of urine into the cellular membrane of the scrotum and penis. Upon examining him, an ulcerated opening was discovered at the root of the penis; the scrotum was enormously distended and of a dull red colour: upon the left side of it there was a gangrenous spot, about two inches in length. His appearance was miserably wretched and emaciated, and his countenance was marked with frightful anxiety; his pulse was very frequent and feeble; his tongue parched and covered with a fur of a dark brown colour; the belly was tender when pressed; and the bladder was distended, and had risen above the pubes.

The history he gave of the case was as follows: three years back he had chancres on the penis, attended with phymosis, and a large portion of the prepuce sloughed away: from this time he dates the origin of the disease in the urethra, as he was shortly afterwards attacked with retention of urine, from which he was relieved by the introduction of a catheter. He has since had repeated attacks of the complaint, which he always succeeded in relieving by means of warm fomentations, &c., until about three weeks previous to his admission at the hospital, at which time his complaint again came upon him, and he found much more difficulty in relieving himself than usual by means of his old remedies, but he at last succeeded in emptying the bladder, the urine passing only by drops. He shuffled on in this way for upwards of a fortnight, without any medical assistance, till, on the seventeenth day from the commencement of this last attack, he suddenly experienced, while straining to make water, a sensation as if he were emptying his bladder, but no urine flowed, and shortly afterwards he perceived that the scrotum was greatly distended. For some little time after this he felt quite easy, but was soon attacked with a violent burning pain in the distended scrotum. The next day he became much worse; the bladder was again filled, and he was in great torture. After much procrastination, a medical gentleman in the neighbourhood was sent for, and, by his desire, he was conveyed to the hospital. He stated that he had, for several years, lived a gay and dissipated life, and scarcely ever enjoyed what might be called perfect health: this his appearance certainly confirmed, as, although in the prime of life, he had the countenance and appearance of an emaciated old man.

As it was perfectly impossible, from the swollen and half-sloughy state of the penis, to introduce a catheter by the urethra, Sir Charles Bell, by whom he was seen immediately on his admission, determined to puncture the bladder, and thus relieve him of one source of irritation, and then to lay open the diseased scrotum. The patient was drawn to the edge of the bed, and the bladder was punctured through the rectum by means of the long-curved trocar, and about a pint and a half of urine escaped. The canula was confined in the bladder by means of tapes and a T bandage. The sloughy portion of the scrotum was freely divided with a bistoury, and a quantity of fetid urine, mixed with pus and mucus, escaped.

The patient being very low and exhausted at the time of the performance of the operation, some brandy was given him, and he had thirty drops of laudanum in camphor mixture shortly afterwards. He soon expressed his relief from suffering, and in the course of the afternoon had several hours' good sleep. A gum elastic catheter was introduced into the bladder through the canula, and the urine continued to dribble away through it. Ordered Hydrar. Submur. gr. i. 4ta quâque horâ: to have brandy and water occasionally.

The day after the operation, he was evidently much improved; his tongue was cleaner, and he had in some measure got rid of the anxious appearance of countenance; the scrotum was much diminished in size, and the sloughing was not spreading. The catheter was withdrawn through the canula, was cleaned and replaced. As he had as yet had no evacuation from the bowels, the canula had not been at all disturbed in its situation. He continued thus to improve daily; the distended scrotum and penis returning to a more natural appearance; the gangrenous portions separated, and a healthy suppuration was established; his appetite improved with the assistance of a slight tonic mixture, and he was allowed six ounces of wine daily. The catheter was repeatedly withdrawn, cleaned, and reintroduced through the canula, which was easily retained in its proper situation by a little attention.

In the course of a few days, the swelling and inflammation had so far subsided as to allow of the introduction of a catheter by the urethra. The instrument passed easily as far as the root of the penis, just anterior to the point where the ulcerated opening was situated: here it seemed entangled, and the incision which had been formerly made in the scrotum was continued up to the point of the catheter, and then, by slightly elevating the point of the instrument, it passed on into the bladder. It was then secured in its position by tapes, and the canula was withdrawn from the rectum. During the evening the patient complained of great pain and irritation in the testicles and urethra, no doubt produced by the catheter; but as the urine passed freely through it, it was not withdrawn. An opiate was given him, and he had a good night's rest. In a day or two the irritation from the catheter had entirely subsided; the water continued to pass occasionally by the rectum, but more frequently through the catheter or beside it. On the 23d May he had a violent attack of diarrhœa, but which, however, was quickly subdued.

He thus continued to improve as rapidly as could be expected: his appetite was good; he was free from pain; and the catheter having been withdrawn, he could make water freely by the urethra; the wounds in the penis and scrotum granulated, and no water had passed by the rectum for some time: in fact, he was considered nearly convalescent, when all at once, without any previous symptoms of indisposition, he, on the morning of the 22d June, became suddenly slightly delirious, and the most alarming symptoms of debility came on: the most energetic means were adopted to combat this sudden state of collapse, but in spite of the most constant attention and care he continued sinking, and died at eight A.M. on the following day; that is, in less than twenty-four hours from the commencement of this last attack.

*Sectio cadaveris.* Upon opening the thorax, adhesions of the pleuræ, and other symptoms of old disease, were apparent; and, upon cutting into the lungs, several large vomicæ were found in them. The viscera of the pelvis were carefully removed, and separately examined. Upon opening the bladder, the inner coat of it was found to be deeply ulcerated: it was of a blackish grey colour, and had a sloughy appearance; the wound made by the trocar in puncturing it had not healed, and was situated nearly close to the entrance of the right ureter. The prostate gland was completely disorganised, and its place was occupied by an abscess which extended backwards about three inches, between the bladder and the rectum. The urethra was unobstructed throughout its whole course, and a staff of a large size could be passed without difficulty.

*Retention of Urine from Inflammation and Swelling of the Prostate Gland.*

John Taylor, twenty years of age, a remarkably athletic and powerful man, was brought to the Middlesex Hospital at eleven o'clock in the evening of the 17th November, 1829, labouring under a severe attack of retention of urine: he was accompanied by a medical gentleman, who gave me the following history of his case. For several days back the patient had been suffering from gonorrhœa, for which complaint he had been furnished with the usual remedies, and was apparently getting better till this morning, when he was suddenly seized with retention of urine: he immediately sought professional assistance, and an attempt was made to introduce a catheter, but without success, and it appeared to add considerably to the irritation. Every auxiliary assistance that could be suggested was adopted, but not a drop of urine would pass. The poor fellow was bled, both generally and by leeches; fomentations were applied to the abdomen and perineum; and opiates and diuretics administered very liberally. Towards night the symptoms became so alarming that, conceiving it would not be possible to get through the night without imminent hazard, he had him conveyed to the hospital, in order to have him relieved by an operation.

When I first saw the patient, he was lying in bed, rolling about in agony, and the bladder was risen above the pubis, greatly distended, and acutely painful on the slightest pressure. In answer to my questions, he told me that he had never before had an attack of retention of urine, and he had always made water in a large stream. The scalding and irritation from the gonorrhœa had been very severe. I learnt from his medical attendant that, in his attempts to pass a catheter, he had always been stopped, as it appeared to him, just anterior to the prostate. Judging from these circumstances, I imagined that the obstruction must arise from inflammation of the prostate gland and neck of the bladder; and taking a large-sized silver catheter, I bent it at the extremity so as to form a very sharp curve, and, having previously warmed and oiled it, I passed it into the urethra, and found I was unable to pass it beyond the prostate: however, I did not withdraw it, but, holding the instrument in the right hand, I introduced the fore-finger of the left hand into the rectum, and could feel the prostate swollen, and it was so acutely tender that, upon making the slightest pressure upwards, the patient roared out with the pain. I then gently elevated the point of the catheter, and, assisting it a little with the finger, it slipped into the bladder. The urine gushed out with such force as to be thrown nearly across the ward, and I think the quantity could not have been much less than three pints. The patient was instantly relieved, and the catheter was withdrawn; as I had no apprehension of any further difficulty in introducing it, and, by remaining, it would have been a source of irritation. A warm bath was got ready; the patient was placed in it for a quarter of an hour; he was then sent to bed, and an opiate was given him. He had a good sleep, and had no return of the attack afterwards: by the use of the bath and other remedies, he was able to leave the hospital in ten days.

I have thought the above case worthy of recording, for the purpose of shewing how very alarming an appearance a really very simple affair may assume. Had it but occurred to the gentleman who brought him to me, and who was a most intelligent and able practitioner, to have examined the bladder by the rectum, I am sure he would have instantly discovered the source of the mischief, and I should have seen nothing of the case. See p. 87.

